# A new turn off fluorescent NIR probe for hypochlorous acid and its applications

**DOI:** 10.55730/1300-0527.3725

**Published:** 2025-03-29

**Authors:** Ayten IBRAHIMOVA, Ayşe Nur ÖNEM, Mehmet ALTUN, Emin Ahmet YEŞİL, Aslı BAYSAL, Hasan SAYGIN, Ahu SOYOCAK, Mustafa ÖZYÜREK

**Affiliations:** 1 Division of Analytical Chemistry, Department of Chemistry, Faculty of Engineering, Istanbul University-Cerrahpaşa, İstanbul, Turkiye; 2 Division of Organic Chemistry, Department of Chemistry, Faculty of Engineering, Istanbul University-Cerrahpaşa, İstanbul, Turkiye; 3 Chemical Technology Programme, Vocational School, Istanbul Gedik University, İstanbul, Turkiye; 4 Department of Chemistry, Faculty of Science and Letters, Istanbul Technical University, İstanbul, Turkiye; 5 Application and Research Center for Advanced Studies, Istanbul Aydin University, İstanbul, Turkiye; 6 Department of Medical Biology, Faculty of Medicine, Istanbul Aydin University, İstanbul, Turkiye

**Keywords:** Hypochlorous acid, NIR, fluorogenic probe, GSH, cytotoxicity

## Abstract

Hypochlorous acid (HOCl) is a potent nonradical oxidant involved in various physiological processes, particularly within the human immune system. In this study, we introduce a novel, rapid, and highly efficient fluorometric method for the detection of HOCl. The method utilizes a near-infrared (NIR)-based fluorescent probe, NIR-QBH, which is characterized by its high sensitivity and chemical stability. NIR-QBH, containing olefinic C=C bonds, exhibits strong NIR emission at 660 nm (λ_ex_ = 618 nm). The detection mechanism relies on the oxidation of the C=C bond in the NIR-QBH structure by HOCl, resulting in the formation of non-fluorescent products. With a detection limit of 0.23 μM, the probe demonstrates a fast response time of 4 min. Glutathione (GSH), an essential biothiol, was employed as a reference HOCl scavenger, and its HOCl scavenging activity was evaluated with an IC_50_ value of 8.97 μM. Furthermore, the developed fluorometric assay was successfully applied for the detection of HOCl in fetal bovine serum (FBS) and aqueous solutions.

## 1. Introduction

Hypochlorous acid (HOCl) is a powerful oxidant with critical roles in several physiological processes, particularly in the innate immune defense mechanisms of the human body [1]. It is synthesized through the myeloperoxidase/halide system in phagocytic cells during respiratory bursts, contributing to the antimicrobial activities of monocytes and neutrophils [2]. However, excessive and unregulated production of HOCl, akin to other reactive oxygen species (ROS), can result in significant cellular damage, manifesting as inflammatory diseases, cardiovascular conditions, arthritis, and other pathologies. This is primarily due to its rapid interaction with sulfhydryl groups in peptides, glutathione-containing proteins, and amino groups, leading to cellular and molecular damage [3].

Although HOCl plays a vital role in various diseases, its functions remain poorly understood due to the limitations of current detection technologies. Conventional methods such as electroanalysis, potentiometry, high-performance liquid chromatography, and chemiluminescence often fail to provide the required sensitivity, selectivity, or spectral resolution for precise and real-time monitoring [4]. In contrast, fluorescence-based approaches offer distinct advantages, including higher sensitivity, superior selectivity, and real-time imaging capabilities [5]. Over time, some fluorescent probes have been developed for HOCl detection [6–8]. However, many of these probes still encounter critical challenges, such as slow response rates, complex synthetic pathways, and short emission wavelengths, which limit their effectiveness in biological and environmental applications. Despite significant advancements in HOCl detection and in vivo imaging, designing highly efficient fluorescent probes for HOCl remains a major challenge. Key priorities include enhancing sensitivity to detect trace HOCl concentrations, developing NIR-emitting probes for clinical applications, and engineering stable, cost-effective fluorophores. NIR fluorescence probes, known for their deep tissue penetration and minimal photodamage, are particularly valuable for clinical applications. Recent studies have focused on hydrazine-based probes, aryl boronic ester probes, and electron-rich fluorophores containing phosphorus (P), selenium (Se), or sulfur (S) atoms. To address existing challenges, Liang et al. (2025) introduced PTZ-BA, a NIR phenothiazine-based dual-responsive fluorescent probe, which demonstrated high selectivity for HOCl, distinct emission signals, a large Stokes shift (275 nm), an ultralow detection limit (11 nM), and a rapid response time of 40 s [9]. Likewise, Zhang et al. (2025) designed NIRF-PA-HOCl, a NIR fluorescence/photoacoustic dual-modal probe that utilizes a spirolactam ring-opening mechanism, achieving 660 nm fluorescence emission, high selectivity, reliable detection limits, and superior dual-modal SBRs [10]. Furthermore, Ma et al. (2025) developed BDP-ENE-Fur-HOCl, a NIR fluorescent probe for real-time imaging of alcoholic liver disease by monitoring HOCl, demonstrating outstanding sensitivity, fast response kinetics, and robust NIR fluorescence at 700 nm for in vitro detection [11]. Nevertheless, there remains a notable gap in the development of NIR fluorescence probes [12] that can facilitate real-time, subcellular-level HOCl detection.

In this context, we synthesized and characterized an NIR-based probe, NIR-QBH, specifically designed for highly selective and sensitive HOCl detection in a fluorescence “turn off” manner. The sensing mechanism involves the oxidation of the C=C bond within the probe structure by HOCl, leading to a decrease in fluorescence intensity. The developed probe exhibits remarkable features, including rapid response, high selectivity, low cytotoxicity, and strong anti-interference capability, making it suitable for detecting HOCl in complex biological matrices.

## 2. Materials and methods

### 2.1. Chemicals and instruments

Sodium hypochlorite (NaOCl), glutathione (GSH), fetal bovine serum (FBS), 3-methyl-2-butanone, phenyl hydrazine, methyl iodide, acetonitrile, ethanol, acetic acid, and 8-hydroxyquinoline-2-carbaldehyde were purchased from Sigma Aldrich (Steinheim, Germany). DMSO and HEPES were obtained from Sigma (USA). Unless otherwise stated, all chemicals used were of analytical grade. UV-Vis spectra were recorded on a Perkin Elmer Lambda 35 spectrometer, and fluorescence measurements were conducted using a VARIAN Cary Eclipse spectrofluorometer (Mulgrave, Victoria, Australia). Elemental analyses were performed using a Thermo Finnigan Flash EA 1112 elemental analyzer. Infrared (IR) spectra were recorded over the range of 400–4000 cm^−1^ on a JASCO FT/IR 4700 spectrophotometer. ^1^H-NMR) spectra were acquired at 500 MHz and carbon-13 NMR (^13^C-NMR) spectra at 125 MHz on a Varian Unity INOVA spectrometer, using CDCl_3_ as the solvent and tetramethylsilane (TMS) as the internal standard. Mass spectra were obtained using electrospray ionization (ESI) on a Thermo Finnigan LCQ Advantage MAX LC/MS/MS spectrometer.

### 2.2. Synthesis of NIR-QBH

**Scheme 1:**
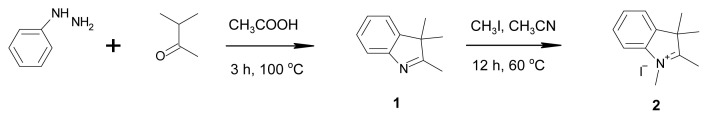
Synthesis of 1-methyl-2,3,3-trimethyl-3H-indolium iodide (2).

**Scheme 2:**
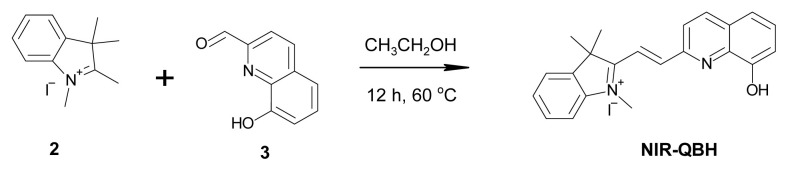
Synthesis of NIR-QBH probe.

The synthesis began with the preparation of 1-methyl-2,3,3-trimethyl-3H-indolium iodide (2). A mixture of phenyl hydrazine (74 mmol, 7.3 mL) and 3-methyl-2-butanone (74 mmol, 8 mL) was added to 40 mL of acetic acid and refluxed at 100 °C for 3 h. After completion, water and dichloromethane (DCM) were introduced to transfer the target product into the organic phase. The organic layer, containing the colored product, was separated from the aqueous phase using a separation funnel and transferred into a flask. The solvent was removed through evaporation, yielding 2,3,3-trimethyl-3H-indole (1) as a dark red liquid. Without additional purification, 150 mL of acetonitrile and 150 mL of methyl iodide were added to the obtained compound (2), and the reaction was refluxed at 55 ºC for 6 h. After the reaction, the flask was cooled in a controlled environment to promote precipitation. The resulting solid product was filtered and dried. To improve yield, the reaction was repeated three times, producing the same compound (2) each time. The final salmon-colored crystalline solid was obtained with a 65% yield (13 g) (Schemes 1 and 2).

**Figure 1:**
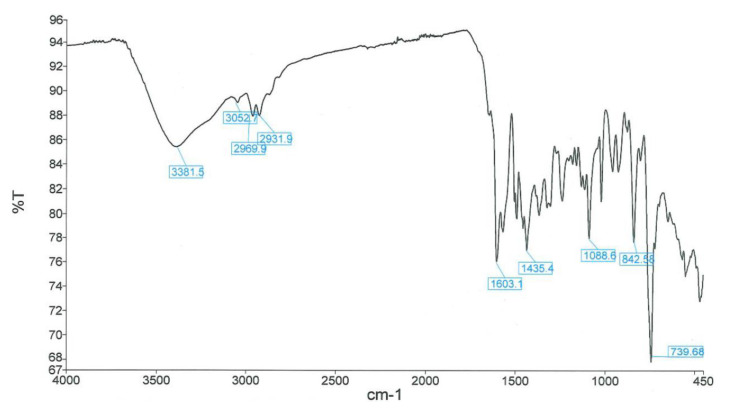
FT-IR spectrum of NIR-QBH probe.

**Figure 2:**
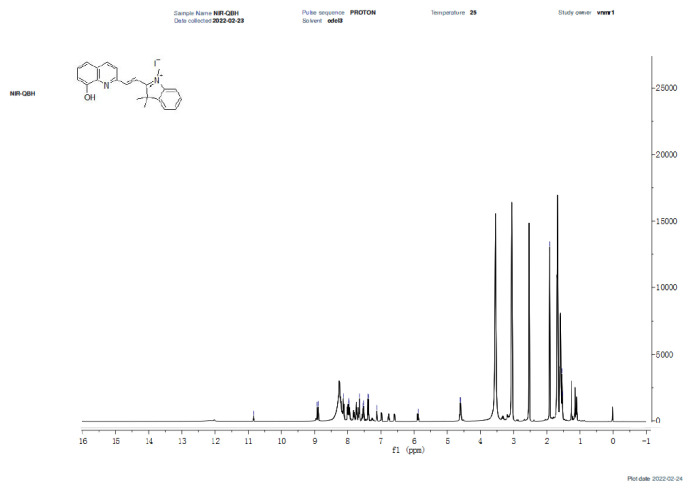
^1^H-NMR spectrum of NIR-QBH probe.

**Figure 3:**
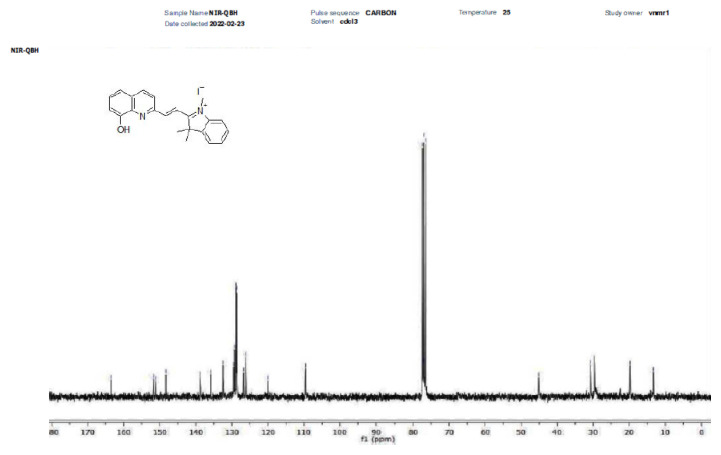
^13^C-NMR spectrum of NIR-QBH compound.

**Figure 4:**
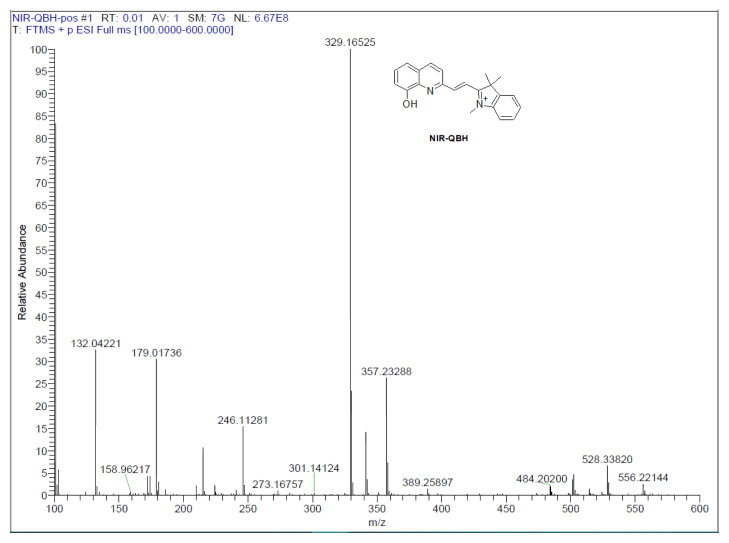
MS spectrum of the NIR-QBH probe.

The synthesis of NIR-QBH was initiated by combining compound 2 (2 mmol, 603 mg) with 8-hydroxyquinoline-2-carbaldehyde (2 mmol, 345 mg) in 40 mL ethanol. The mixture was refluxed at 80 °C for 12 h. Subsequently, ethanol was partially evaporated, and the mixture was stored overnight in a refrigerator. The resulting solid precipitate was filtered and purified via column chromatography (CHCl_3_ – MeOH, 20:1). NIR-QHB probe compound was obtained as a solid product of dark brown color. Yield: 70%, 630 mg. FT-IR (υ, cm^−1^): 3381 (OH), 3052, 2969 (C-H_aromatic_), 2931 (C-H_aliphatic_), 1603 (C=C_alkene_), 1435 (C=N), 1088 (C-OH), 900–700 (plane bending vibrations for benzene rings) (Figure 1). ^1^H NMR (500 MHz, CDCl_3_): δ 11.35 (s, 1H), 10.86 (s, 1H), 8.47 (d, J = 15.7 Hz, 1H), 8.11 (d, J = 8.5 Hz, 1H), 7.77 (d, 2H), 7.46 (m, 2H), 6.37 (d, 1H), 4.61 (d, 1H), 3.14 (s, 2H) 1.86 (s, 6H), 1.37 (t, 3H) (Figure 2). ^13^C NMR (75 MHz, CDCl_3_): δ 13.5, 19.4, 30.2, 30.8, 46.1, 109.3, 109.3, 120.2, 126.1, 126.6, 126.7, 128.4, 128.9, 129.3, 129.5, 131.9, 134.5, 137.8, 146.9, 150.8, 152.1, 162.9 (Figure 3). HRMS (+ESI): m/z [M-I]^+^= 329.16525 (C_22_H_21_N_2_OI: 456.31941 g/mol) (Figure 4).

### 2.3. Fluorometric procedure for detection of HOCl

A stock solution of HOCl (1.5 mM) was prepared in distilled water, with the concentration verified spectrophotometrically (ɛ_HOCl_ = 100 M^−1^ cm^−1^) at 235 nm. NIR-QBH stock solutions (1.0 mM) were prepared in ethanol containing 8% DMSO. For the fluorometric procedure, 0.1 M HEPES buffer (1 mL, pH = 7.4), 0.15 mM HOCl (0–0.3 mL), and 1.0 mM NIR-QBH (2 mL) were mixed and diluted with EtOH to a final volume of 4.0 mL. The mixture was incubated at 25 °C for 4 min, and fluorescence intensities were recorded at λ_em_ = 660 nm.

### 2.4. RP-HPLC-PDA analysis of NIR-QBH

The synthesized probe was analyzed using the RP-HPLC-PDA method. For these analyses, a Waters Breeze™ 2 Model HPLC system (Milford, MA, USA) was used, which included a 1525 model binary pump, a 2465 model photodiode array detector (PDA) (Chelmsford, MA, USA), a Hamilton 25 μL syringe (Reno, NV, USA), and an Zorbax C18 analytical column (4.6 mm × 150 mm, 5 μm) (Milford, MA, USA). A gradient elution method was applied to determine the initial amount of the probe and the amount of unreacted probe after reacting with HOCl. The mobile phase consisted of methanol (A) and 1.0% (v/v) acetic acid (B). The gradient elution conditions were as follows:

- 1st minute: 40% A – 60% B (Gradient slope 1.0)
- 5th minute: 60% A – 40% B (Gradient slope 1.0)
- 8th minute: 80% A – 20% B (Gradient slope 1.0)
- 13th minute: 90% A – 10% B (Gradient slope 1.0)
- 15th minute: 70% A – 30% B (Gradient slope 1.0)
- 20th minute: 50% A – 50% B (Gradient slope 1.0)

The sample injection volume was 20 μL, the flow rate was 0.8 mL/min, and the detection wavelength was 395 nm with a total elution time of 20 min. This method was adapted from an existing HPLC method used for determining HOCl scavenging activity in biological samples, as reported in the literature [13].

### 2.5. Cytotoxicity assays

For cell culture, The Dulbecco’s Modified Eagle Medium (DMEM)-10% heat-inactivated fetal bovine serum) with 1% penicillin/streptomycin (Diagnovum, Ebsdorfergrund, Germany) was used and A549 cells were incubated at 37 °C in a humidified atmosphere and 5% CO_2_. A549 cells were seeded into 96-well plates at a density of 1 × 10^4^ cells/well and incubated 24 h. Then, probe NIR-QBH (0–1.0 mM) and control (no probe) were added to the microplate and exposed for different times (24, 48 and 72 h). Each concentration of the probe NIR-QBH was freshly prepared at the intended concentration and vortexed for 4 min.

The MTT method was utilized to measure the cellular cytotoxicity. The cytotoxicity of the A549 cells by mitochondrial activity after exposure to different concentrations of the probe NIR-QBH was evaluated using an MTT test kit (Elabscience, E-CK-A31, USA). After 24, 48, and 72 h, the medium was changed with fresh medium (100 μL), and MTT working solution (50 μL, 5 mg/mL) was added to each well. The media was replaced with 150 L of dimethyl sulfoxide (DMSO) (Tekkim, Türkiye) after 3 h of incubation at 37°C with 5% CO_2_ for dissolving the formazan [14,15]. Lastly, absorbance of each well was measured using a UV-VIS microplate reader at 570 nm (Thermo Scientific, Multiskan GO, USA).

The antioxidant capacity of cell lysates was measured by the CUPRAC method [14,15]. A portion of the control and probe NIR-QBH-treated cells was used to assess antioxidant activity and oxidative stress in the cells. The cell mixture (control and probe NIR-QBH-treated cell portions) was mixed with CuCl_2_ (0.01 M), Neocuproine (7.5 × 10^−3^ M), and CH_3_COONH_4_ (1.0 M). After the 30-min incubation, the absorbance was measured at 450 nm with a UV-VIS microplate reader (Thermo Scientific, Multiskan GO, USA). Protein content in the cell lysates were tested using the Bradford assay [14]. The samples and control were mixed with diluted Bradford reagent and samples into the wells of a 96-well plate. The mixtures were incubated for 15 min at room temperature and absorbance were read at 595 nm with a UV-VIS microplate reader (Thermo Scientific, Multiskan GO, USA). Neccessary reagent blank applications in each parameter and control application (without probe NIR-QBH cells) was used. All experiments were performed at least in triplicate.

### 2.6. Statistical analysis

The differences between the control and samples were analyzed using ANOVA with a post hoc Tukey test (p < 0.05).

## 3. Results and discussion

### 3.1. Sensing mechanism of NIR-QBH to detect HOCl

Several fluorescent probes have been developed for HOCl detection, utilizing the oxidation of C=C double bonds as a key mechanism. This process typically involves the formation of an epoxide, followed by ring opening and bond cleavage, which has been widely documented in the literature [16–19]. Additionally, HOCl-mediated oxidative cleavage of olefinic C=C bonds can lead to the generation of aldehydes and carboxylic acids, among other breakdown products. These species, which are often more stable and non-chlorinated, may arise due to further oxidation of initially formed, less stable chlorinated intermediates. Furthermore, HOCl-induced disruption of molecular conjugation, particularly involving C=C bonds, can cause distinct spectroscopic shifts, including fluorescence intensity variations. Building on this mechanism, numerous fluorescent probes have been designed to exploit these chemical transformations for selective HOCl detection [20–24].

**Figure 5:**
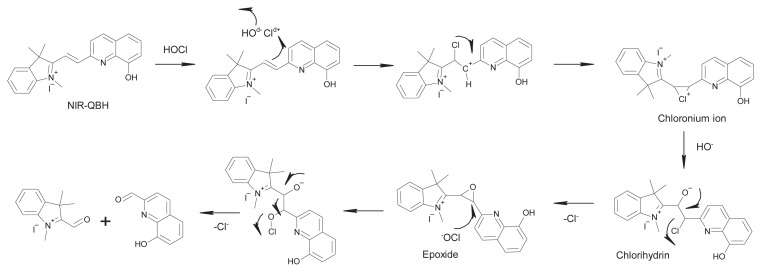
Possible sensing mechanism of NIR-QBH and HOCl.

The sensing mechanism of the NIR-QBH probe is based on the oxidative cleavage of olefinic C=C bonds by HOCl, leading to the disruption of the probe’s conjugation system and the formation of nonfluorescent products. This mechanism involves the initial oxidation of the C=C bond to form an epoxide, followed by ring-opening and bond cleavage reactions. These changes result in a decrease in fluorescence intensity, characteristic of a “turn off” response (Figure 5).

**Figure 6:**
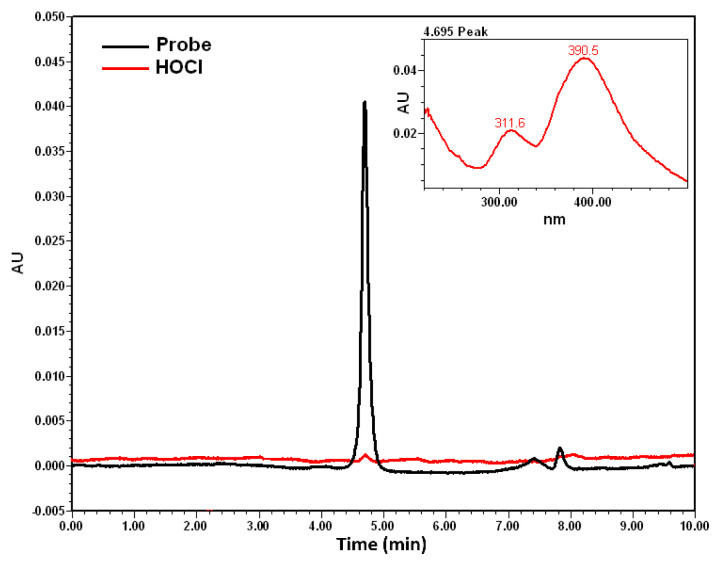
HPLC-PDA chromatograms of the probe and HOCl at 395 nm.

**Figure 7:**
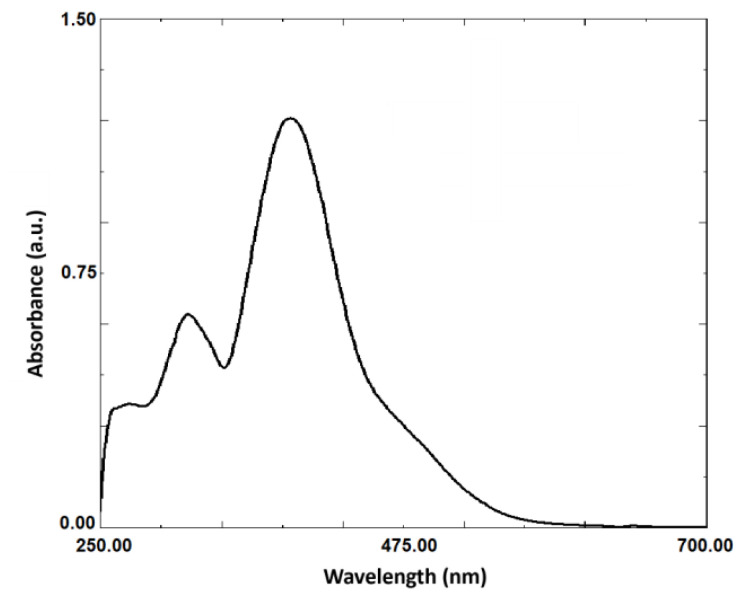
UV-Vis spectrum of the probe.

**Figure 8:**
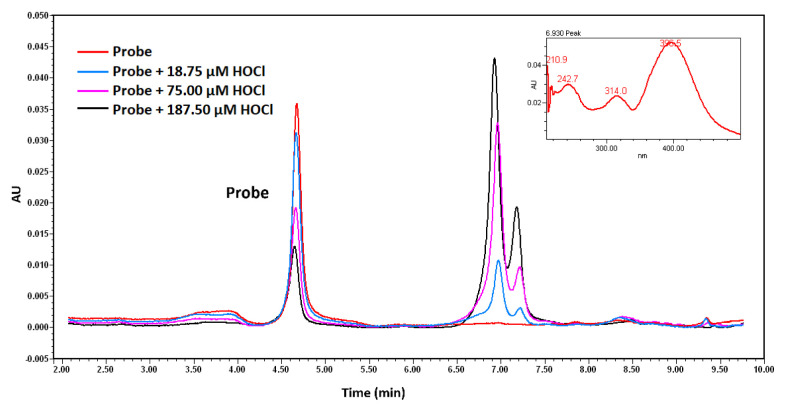
Chromatograms of the probe (c_final_ = 5 × 10^−5^ M) with the addition of different concentrations of HOCl in mixture solutions.

When examining the chromatogram of the probe (Figure 6), a peak at 4.67 min was observed for the probe (c_final_ = 5 × 10^−5^ M), and the PDA spectrum was included in the figure. A comparison of this spectrum with the UV-visible spectrum (Figure 7) revealed a similar spectral pattern. Additionally, Figure 6 showed that HOCl did not exhibit a peak at 395 nm. Different concentrations of HOCl were added to the reference medium containing the probe, and the corresponding chromatograms are presented in Figure 8. As seen in Figure 8, increasing the HOCl concentration resulted in a decrease in the peak intensity of the probe, along with the appearance of peaks corresponding to reaction products. This confirms that the probe reacts with HOCl, as observed in the developed fluorometric method.

### 3.2. Spectroscopic properties of NIR-QBH toward HOCl

**Figure 9:**
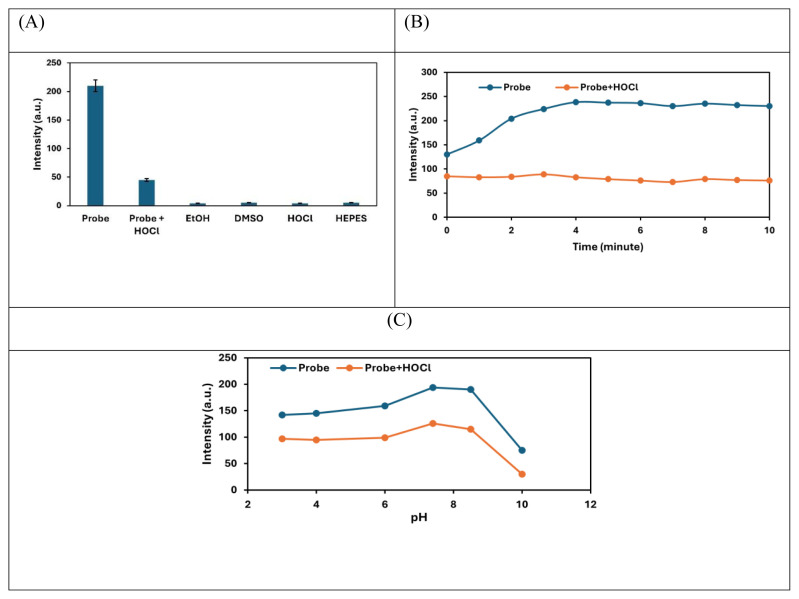
(A) Fluorescence intensities of the probe in the presence of other components present in the reaction mixture. (B) Intensities versus incubation time and (C) pH curves of NIR-QBH probe (1.0 mM) alone and in the presence of HOCl (1.5 mM).

The fluorescence intensity of the NIR-QBH probe was evaluated in the presence of various components in the reaction mixture at λ_em_ = 660 nm. A significant fluorescence decrease was observed exclusively in the presence of HOCl, confirming the probe’s selectivity (Figure 9A).

The stability of the NIR-QBH probe and its response time to HOCl were also assessed. The probe demonstrated excellent stability over 4 min of testing, with an immediate and stable response upon addition of HOCl (Figure 9B). The rapid response (4 min) of the probe highlights its utility for real-time HOCl detection. The optimal pH for fluorescence detection was determined to be 7.4, corresponding to physiological conditions (Figure 9C). This also shows that the detection of HOCl in the organism can be made with the developed probe under physiological conditions.

**Figure 10:**
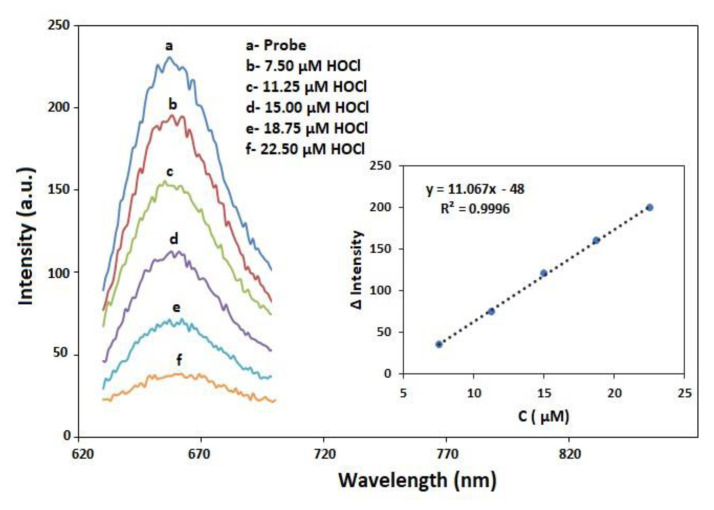
Emission spectra of NIR-QBH (1 mM) with increasing concentrations of HOCl (0–22.50 μM). Inset: calibration curve between the δI values of NIR-QBH and HOCl concentration.

The emission intensity of NIR-QBH decreased proportionally with increasing HOCl concentration, enabling sensitive and selective detection (Figure 10). It showed good linearity in the range of 7.5–22.50 μM (r = 0.9996, N = 5), with the limits of detection (LOD = 3_sbl_/m) and quantification (LOQ=10s_bl_/m) values of 0.23 and 0.77 μM, respectively (m: regression slope, s_bl_: standard deviation). The system is nearly fully quenched at 22.50 μM HOCl (linear range), and complete non-emissive behavior is expected at approximately 25–30 μM HOCl. The intra- and interday precisions (RSD, % values) were found as 1.86 and 2.91, respectively.

Detection limits and HOCl fluorophores (NIR-based fluorogenic probes) in the literature are given in Table [12]. In the literature, there are a limited number of HOCl probes with emission in NIR regions [25–30]. When the LOD values of these probes, which are used for different HOCl bioimaging models, are compared, it is seen that HOCl detection is performed with comparable sensitivity in the newly developed method.

### 3.3. Specificity of NIR-QBH toward HOCl

**Figure 11:**
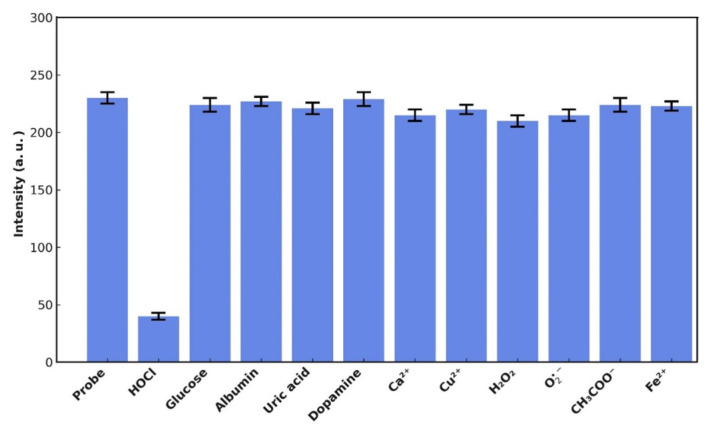
Potential interference effect of some common bioactive substances.

The specificity of the NIR-QBH probe was evaluated in the presence of potential interferents, including glucose, albumin, uric acid, dopamine, Ca(II), Cu(II), Fe(II), CH_3_COO^−^ and H_2_O_2_. Fluorescence changes were observed only in the presence of HOCl, demonstrating the probe’s high selectivity in complex biological systems (Figure 11).

### 3.4. Detection of HOCl in FBS in the prescence of GSH

**Figure 12:**
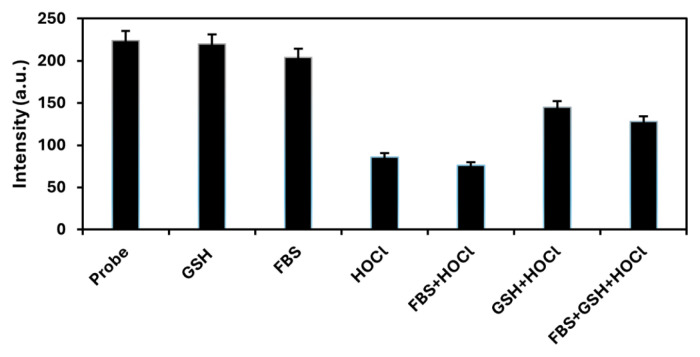
Fluorescence intensities of mixtures of NIR-QBH probe (1.0 mM) formed in different combinations with GSH (1.0 mM), FBS (1:10 diluted) and HOCl (1.5 × 10^−4^ M) (N = 3).

To assess the applicability of the NIR-QBH probe in complex biological systems, its fluorescence response was tested in FBS. The intensity changes of “NIR-QBH probe + HOCl” in FBS were consistent with those in aqueous solutions [31]. Upon addition of GSH (1.0 mM), HOCl scavenging activity was calculated as 40.62% in aqueous solutions and 44.02% in FBS, demonstrating the probe’s effectiveness in biological matrices (Figure 12).

### 3.5. HOCl scavenging activity of GSH

**Figure 13:**
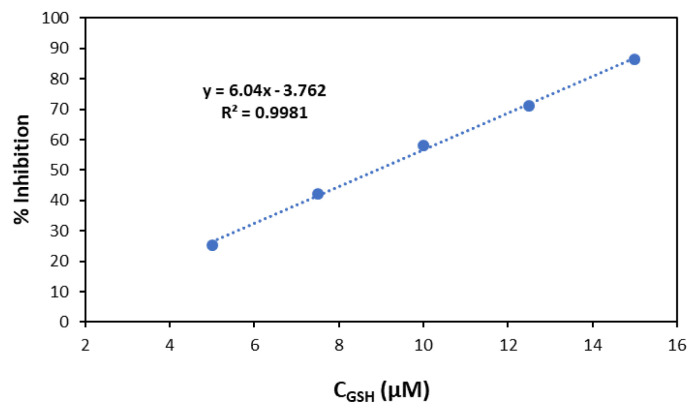
Concentration-response inhibition (%) for GSH (1.0 mM) according to the fluorometric assay.

**Figure 14:**
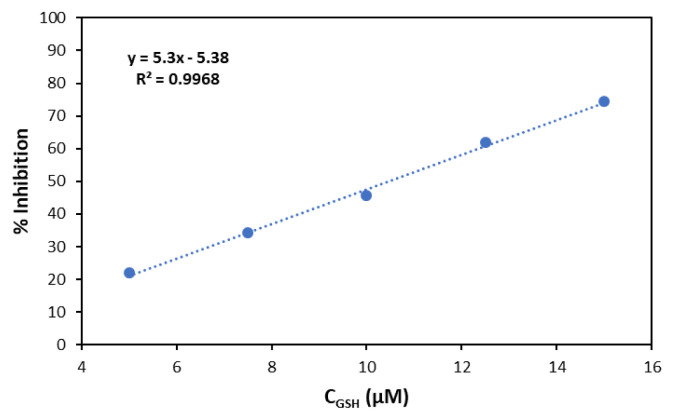
Concentration-response inhibition (%) for GSH (1.0 mM) according to the resorcinol assay.

GSH, a key thiol type antioxidant, reacts with HOCl to form GSCl and water [32**]**. The scavenging activity of GSH was determined via competitive kinetic assays using NIR-QBH and a reference resorcinol assay. The HOCl scavenging activity of GSH was determined using both fluorometric and resorcinol assays, yielding IC_50_ values of 8.91 ± 0.44 μM and 10.44 ± 0.76 μM, respectively, consistent with literature values (Figures 13 and 14). Özyürek et al. (2012) seemingly reported GSH as an HOCl scavenger displaying an IC_50_ value of 8.95 ± 0.47 μM [13].

### 3.6. Cytotoxicity

**Figure 15:**
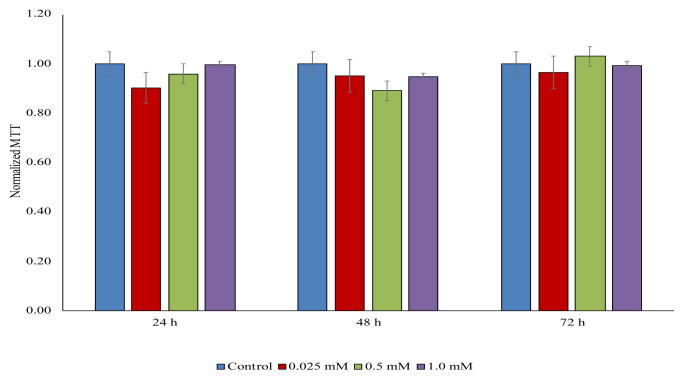
Concentration-response cell viability graph for NIR-QBH probe. The result was presented using normalized MTT of A549 cells treated with probe NIR-QBH at 0.025 mM, 0.5 mM, and 1.0 mM and A549 cells without probe NIR-QBH (control).

The potential toxicity of NIR-QBH in A549 cell lines was tested with an MTT assay. As illustrated in Figure 15, the cell viability after 24 h, 48 h, and 72 h exposure of NIR-QBH to A549 cells at studied concentrations (0.025 to 1.0 mM) was not significantly changed (p < 0.05). However, in some cases, MTT levels declined with an insignificant input. These results indicate that NIR-QBH shows low cytotoxicity and good biocompatibility. Various probes were tested for cytotoxicity using different cell types, including A549, Rma-1, SH-SY5Y, PC-3, and HeLa [33–35]. Li et al. (2013) examined NIR fluorescent probe 7-[(5-Nitrofuran-2-yl)methoxy]-3H-phenoxazin-3-one, and tested toxicity using standard MTT assay at 24 h exposure on Hela cells [33]. The result showed that the probe was not significantly changed the viability of cells between the probe concentrations at 1–20 μM on the viability of. Shang et al. (2021) investigated the cytotoxicity of NIR fluorescent probes by e 3-(2-chlorophenoxy)propan-1-amine derivatives on HeLa cells using MTT assay, and no significant differences were found even at 10 μM of the probe concentration [34]. In another study, NIR fluorescent probes using dihydroxanthene derivatives were tested on NIH-3T3, RTE, SH-SY5Y, and PC-3 cells for 24 h at different concentrations of the probes (1–50 μM), and no significant inhibition was observed up to 20 μM concentration of probes [35]. In our study, we tested higher concentrations of the NIR probe with various exposures. The findings indicated the remarkable biocompatibility with longer exposures compared to literature.

**Figure 16:**
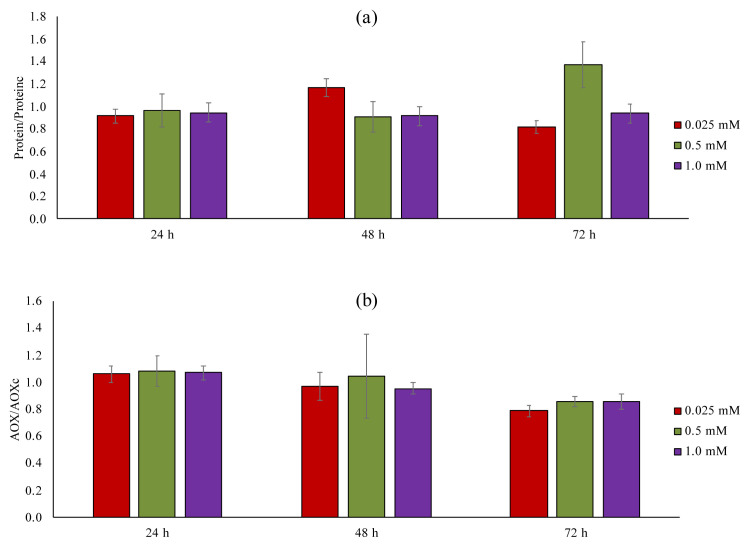
(a) Protein response by Bradford assay and (b) Antioxidant response (AOX) by CUPRAC assay during 24-, 48-, and 72-h exposure of NIR-QBH. The presentation was calculated using protein content of the cells treated with probe NIR-QBH (Protein) to control cells without probe NIR-QBH (Proteinc), and antioxidant activity of cells treated with probe NIR-QBH (AOX) to control cells without probe NIR-QBH (AOX_C_)

To understand the adverse effect of chemicals in complex biological systems, the protein content of the cells was examined as a biomarker (Figure 16a) [36]. Except for 72 h exposure, there were no significant differences between the NIR-QBH concentrations at 24 h and 48 h exposures and controls (p < 0.05). These results indicated the limited protein expression and not easily influenced by exogenous substances since protein levels provide insight into the biomolecular pathway of any chemicals [37,38].

Oxidative stress, owing to an antioxidant imbalance, is known as mediator of cell damage pathways. Therefore, antioxidant activity was also examined [39]. Figure 16b depicts the antioxidant activity of A549 cells exposed to NIR-QBH in different concentrations and different incubations. The antioxidant responses of A549 cells were not significantly changed after expose to NIR-QBH in different concentrations and different incubations (p < 0.05).

## 4. Conclusions

In this study, a novel near-infrared (NIR)-based fluorescent probe, NIR-QBH, was developed for the rapid and selective detection of hypochlorous acid (HOCl). The sensing mechanism relies on the oxidation of olefinic C=C bonds within the probe’s structure by HOCl, resulting in the disruption of the conjugated system and a fluorescence “on-off” response. This method offers several advantages, including high sensitivity, selectivity, low cytotoxicity, and rapid response time (4 min). The detection limit (LOD) of 0.23 μM demonstrates the probe’s suitability for detecting HOCl at biologically relevant concentrations. Glutathione (GSH) was used as a reference compound to evaluate the HOCl scavenging activity, yielding results consistent with those in the literature. The fluorometric assay successfully quantified HOCl scavenging activity in both aqueous and fetal bovine serum (FBS) matrices, confirming its applicability to complex biological systems. The cytotoxicity tests conducted in A549 cell lines revealed that the NIR-QBH probe exhibits low cytotoxicity and good biocompatibility, further enhancing its potential for biological and clinical applications. Future research may focus on employing the NIR-QBH probe for fluorescence imaging to elucidate the role of HOCl in cellular and pathological processes. This work contributes to the advancement of HOCl detection technologies and paves the way for further applications in biomedical research and clinical diagnostics.

## Figures and Tables

**Table:** Detection limits (LOD) and Fluorophores of some NIR-based fluorogenic probes.

Probe	Fluorophores	λ_em_ (nm)	LOD (μM)
ref.^25^	Rhodamine	650	0.03
ref.^26^	Naphthalimide	620/630	0.17
ref.^27^	Coumarin	685	0.17
ref.^28^	Coumarin	660	0.12
ref.^29^	Coumarin	650	0.05
ref.^30^	Nil Red	650	4.37
This work	Cyanine	660	0.23
